# Sponge Grafting

**Published:** 1885-03

**Authors:** Edward C. Briggs

**Affiliations:** Boston, Mass.


### SELECTIONS.

### Sponge Grafting.

BY EDWARD C. BRIGGS, M.D., BOSTON, MASS.

Read in Section of Oral and Dental Surgery, American Medical Association, May, 1884.

In 1879, Mr. D. J. Hamilton, lecturer on pathology, the School of Medicine, Edinburg, prepared a paper "On the Process of Healing." which appeared in the *Journal of Anatomy and Physiology*, Vol. xiii. He there made the statement and proved experimentally that in a granulating surface there were no new vessels formed, but that the superficial capillaries of the part were pushed upward, as granulating loops, by the action of the heart; the projection being permitted from the fact that the restraining influence of the skin had been removed.

Two years later, in the November number of the *Edinburgh Medical Journal*, Mr. Hamilton appears with a communication on "Sponge Grafting."

He there states that while getting the information for his former paper, he was led to believe that the process of vascularization, as seen on a granulating surface similar to that which occurs when a blood clot or a fibrinous exudation is replaced by a vascular cicatricial tissue, and that the blood clot or fibrinous lymph acts simply mechanically to give support to the projecting loops of capillaries. With this idea he searched around for something artificially to replace the blood clot, and hit upon sponge. This, he reasoned, was a porous tissue which would imitate the interstices of the fibrinous net-work of a blood clot. Being animal tissue it would, like cat-gut or the blood clot, be absorbed under favorable circumstances. I will not go into the details of his experiments, although they would be found very interesting. Suffice it to say that his experiments justified his theories.

He first experimented with the well known chronic ulcers of the leg. A piece of sponge, prepared as I shall describe later, was fitted to the sore, the edges of the sponge being tucked in under the indurated edges of the ulcer. Over this some simple antiseptic dressing with bandage.

Dressed on the following day the sponge was found partly filled with purulent discharge. On dressing the second day, there was *distinct putrefactive odor*, and the sponge was washed with carbolic solution. The sponge then appeared slightly red at the most shallow parts, and the edges of the wound reached farther inwards over the sponge.

The third day the sponge was beginning to adhere to the granulating surface, and at one point looked as though it were beginning to dissolve. The fifth day the thinnest parts of the sponge were growing hard and seemed to be filling with organizing tissue; picked at these parts it bled freely, showing that the blood vessels had already begun to climb up through its pores.

The growth was slow, but at the end of three months only a small piece of the sponge was to be seen. The sponge, when originally fitted to the part, was five inches in diameter.
One month later, four months from beginning, Mr. Hamilton showed the patient to the Medico-Chirurgical Society, with no traces of the sponge to be seen, and a healthy granulating surface of only an inch and a half diameter.

This, the first experiment, was satisfactory in every way, proving the theory correct, and showing that sponge would act even better than blood clot; for while a blood clot would have been destroyed by the putrescent condition of the wound, the more resistant sponge did not seem to be at all affected.

Since Mr. Hamilton's paper, surgeons all over the world have been experimenting with sponge-grafting, and many successful cases have been reported from time to time. Seeing these reports, the idea suggested itself to me that morbid conditions in the mouth, associated with loss of the soft tissues, might also be benefitted by the use of sponge.

In giving my experiments I will state just what I did, and not what I should now do after the experience I have had. It happened that a patient of mine, Mr. N. G. B., had been annoyed for three years by a deep depression over the roots of the right superior first bicuspid, the result of a severe periostitis. The chief cause of annoyance was that the food was collected there and the pus retained. It was not a simple fistula, but a cavity ½ inch deep, and 3-16 of an inch in diameter. I had tried in various ways to restore the lost tissue, or at least to narrow the opening down to a small fistula. I had made stimulating applications, had created a fresh blood clot to fill the cavity, hoping it might organize, but all to no purpose; in fact, the cavity tended to grow larger, until I thought of trying sponge.

January 26, 1884, I fitted to the cavity a piece of sponge, previously prepared according to formula number one of Dr. Edward Borck, of St. Louis. The formula is as follows: Take a fine sponge, soak it for three or four days in a 20 per cent. solution of hydrochloric acid; squeeze dry, soak for ten days in a solution of iodoform in ether (ʒi—℥i); evaporate ether, and keep in an air-tight vessel.

Having fitted a piece of sponge thus prepared I dismissed the patient.

The next day I saw him, and not expecting any success, I attempted to pull out the sponge. To my surprise it resisted my efforts. I persisted and pulled it out, disclosing a fresh granulating surface which bled freely.

I packed with fresh sponge. The next day I again pulled out the sponge, meeting with the same resistance. The appearance was the same as the day before, excepting that the space had partially filled up with fresh granulations.

This treatment was pursued for two months, after the first week Mr. B. coming only twice a week. The sponge would apparently become attached for a day or two, and then would work out, necessitating the application fresh sponge. I never again got so good an adhesion as after the first piece was put in, and at no time did there seem to be evidence that the sponge would organize; but, either as a stimulant or a support to the granulations, it accomplished what I had failed to do with other means.

The patient now presents simply a small opening admitting a probe about three-sixteenths of an inch. He is not now annoyed by the collection of food or the retention of pus.

As I have said, I had previously tried the blood clot without success, for, owing to the putrescent condition of the sore, it would always come away in a short time without any change in the cavity being noticeable. With the sponge it was different. The discharge went on through the sponge, the sponge remaining in position and giving support to the granulations which were not in direct line of the discharge.

Case II. About the 1st of April, encouraged by the success of my first experiment, I undertook another case.

This patient, Mr. J. B., relates that three years ago, after a separation had been made between his right superior first and second molars, he began to have trouble in the gum. About two years ago, while in Honolulu, the annoyance was so great that he consulted a dentist, who lanced deeply down between the teeth. He forced the lancet nearly at the roof of the mouth, and drawing it through between the teeth, to the cheek. Where this cut healed the parts contracted, making the trouble worse. It did not heal, however, between the teeth, and when I saw the patient a year ago, there was a deep depression between the teeth. The gum bled freely on being touched. The annoyance was great-amounting sometimes to severe pain. I suggested contouring the teeth to protect the gum, ' ut as the teeth were both very sensitive and the operation would necessitate a great deal of cutting, the patient declined.

When, the 1st of April, in response to a note from me, I examined him with a view to trying sponge, I found the trouble worse than ever, the hollow between the teeth at one part extend, ing two-thirds up the roots of the teeth.

I began the treatment on the 7th of April. After wiping the surface of the gum with deliquesced crystals of carbolic acid, and scraping off the slough, I had a fresh, healthy surface to act upon.

I had prepared for this case a sponge in the following manner: I first cleaned it of its calcareous salts by washing in dilute aqua regia, then allowed it to remain for twenty-four hours in a dilute solution of tincture of iodine, squeezed it dry and put it in a saturated aqueous solution of boracic acid, ready for use.

To protect the gum and prevent the dislodgment of the sponge, I had previously prepared a piece of hard rubber made to arch over the opening between the teeth, resting upon the gum on either side. Having fitted a piece of sponge to the exposed surface, I painted the gum and the inside of the rubber cap with a solution of resin, and put the cap in position.

In three days I removed the cap, syringed the parts, without removing the sponge, with a 1–200 solution of carbolic acid, and replaced the plate as before.

Three days later I again took off the plate, and this time detached the sponge. It was only attached on one side, but at that point the gum had already grown down a little. On dressing it this time, I returned to the use of sponge prepared as for the first case, having an idea that it worked better. I have seen the patient twice a week up to the present time. The case is progressing very favorably, and the cavity is filling up as rapidly as I could wish.

With the present experience I should now, in treating such cases, first be sure that the parts were thoroughly cleaned. I should have the sponge shaped as accurately as possible, not forgetting to allow for the swelling; for a day or two I should endeavor to keep some pressure upon the sponge to aid in its attachment. After that time I should disturb the sponge as little as possible, simply washing away the discharge with some antiseptic solution. I should wait with patience, not expecting to see great progress in a very short time, and have more faith to believe that if let alone, the sponge would really organize, as in Mr. Hamilton's cases.

In regard to its application, I think it can be applied not only to such cases as I have described, but to all cases where it is desired to reproduce soft tissues. I shall try it, and expect success, in the aggravated cases of the so-called "Rigg's Disease," where there is loss of tissue about the roots or teeth.—*Jour. of the Am. Med. Ass'n.*

